# Dietary intakes of cysteine, glutamate, proline, and tryptophan are associated with hypertension risk in Chinese children and adolescents: a national cross-sectional study integrating machine learning

**DOI:** 10.3389/fnut.2026.1798822

**Published:** 2026-04-30

**Authors:** Lianlong Yu, Hongwei Wang, Zhimin Lv, Changqing Liu, Yiya Liu, Meina Tian, Qianrang Zhu, Zhenchuang Tang, Yao Chen

**Affiliations:** 1 Shandong Center for Disease Control and Prevention, Jinan, China; 2 Department of Health Care, People’s Hospital of Rizhao, Rizhao, China; 3 Department of Clinical Nutrition, People’s Hospital of Rizhao, Rizhao, China; 4 Hebei Center for Disease Control and Prevention, Shijiazhuang, China; 5 Guizhou Center for Disease Control and Prevention, Guiyang, China; 6 Jiangsu Provincial Center for Disease Control and Prevention, Nanjing, China; 7 Institute of Food and Nutrition Development, Ministry of Agriculture and Rural Affairs, Beijing, China

**Keywords:** adolescents, children, dietary amino acids, hypertension, machine learning, national survey

## Abstract

**Objective:**

The aim of the study was to examine the relationships between dietary intake of amino acids and the risk of hypertension and to find some important amino acids as the targets of intervention in Chinese children and adolescents.

**Methods:**

The 2016–2019 China Children and Lactating Women Nutrition and Health Surveillance (CCLWNHS) was used to draw data and included 12,187 children and adolescents aged 6–18 years. A validated food frequency questionnaire (FFQ) was used to determine dietary intake with a weighing procedure. After the process of feature selection based on the Boruta algorithm, multivariate logistic regression was used to determine the relationships between amino acids and hypertension. The predictive performance was assessed through the development of XGBoost, LightGBM, NB and NN models. The SHapley Additive exPlanations (SHAP) approach was used to identify key features. Stratified analyses, interaction tests, and restricted cubic spline analyses were done as well.

**Results:**

Cysteine, phenylalanine, histidine, glutamic acid, and proline were positively related to the risk of hypertension in the fully adjusted multivariate model (ORs: 2.000, 1.649, 1.387, 1.052, and 1.105, respectively), and threonine, valine, alanine, and tryptophan were negatively related (ORs: 0.587, 0.704, 0.809, and 0.516). LightGBM was the most successful predictive model (AUC = 0.793). SHAP analysis revealed that the core amino acids that affected the risk of hypertension were cysteine, glutamic acid, tryptophan, and proline. Sensitivity analyses showed that the relationships of these amino acids with hypertension were different in the subgroups based on age, sex, school type, physical activity level, and energy intake, and had a linear dose–response relationship.

**Conclusion:**

A greater intake of cysteine, glutamic acid and proline through the diet is linked to a higher risk of hypertension among Chinese children and adolescents but tryptophan is protective. Such results give a scientific foundation on the development of specific nutritional intervention programs and optimization of protein consumption to prevent early hypertension in life.

## 1 Introduction

Hypertension among children and adolescents has become a worldwide health issue (1, 2). It is becoming an alarming threat to the physical and mental well-being of the youth in China as its prevalence is increasing every year (3, 4). Hypertension at an early age may lead to cardiovascular damage in children and adolescents and significantly predispose people to chronic diseases in adulthood, such as hypertension, coronary heart disease, stroke, and kidney disease, thus creating long-term impacts on the health of the individual and healthcare costs to the society (5–9). It is therefore important to determine the risk factors of hypertension that can be modified and formulate specific prevention and control interventions at a young age in order to ensure a healthy life throughout the lifetime.

The pathogenesis of hypertension among this population is not fully known. The dietary habits are one of the main environmental determinants of blood pressure. Although the impact of macronutrient imbalances (protein, fat, and carbohydrate intake) on blood pressure has been widely investigated (10–13), the research on the association between the intake of particular amino acids and the risk of hypertension among children and adolescents is sparse.

The basic components of proteins known as amino acids have recently been identified to have multifaceted roles in blood pressure regulation (14, 15). Indicatively, arginine is a precursor of nitric oxide, which facilitates vasodilation and enhances the performance of the vascular endothelium (16, 17). Amino acids that contain sulfur can also cause vascular endothelial damage due to their effects on homocysteine metabolism (18, 19). Furthermore, the metabolic dysregulation of the aromatic and branched-chain amino acids has been strictly associated with insulin resistance and chronic inflammation and indirectly associated with blood pressure regulation (20–23). These processes indicate that the various kinds of dietary amino acids can affect blood pressure homeostasis through various mechanisms.

Epidemiological data of dietary amino acids and hypertension among children and adolescents is limited despite the presence of mechanistic evidence. The existing literature is predominantly based on adults or targeted groups of patients, so there is a gap in the systematic research of younger groups, especially within the Chinese cohort. The growth and developmental peculiarities of children and adolescents are distinct and significantly different compared to adults in terms of amino acid metabolism, physiological needs, and diets (24). Also, the Chinese nutritional patterns and dietary arrangements contain regional characteristics which significantly differ with the western diets, which is why local studies are required.

This study uses a large scale and nationally representative sample of the China Children and Lactating Women Nutrition and Health Surveillance (CCLWNHS) to systematically investigate the relationships between the intake of dietary amino acids and the risk of hypertension in Chinese children and adolescents. The proposed study will combine the classical epidemiological methods with modern machine learning methods to determine the essential amino acids, which will prove the effectiveness of the targeted nutritional intervention and guide the specific approach to the prevention and control of hypertension during early life.

## 2 Materials and methods

### 2.1 Study design and participants

The current study was conducted on the basis of the data of the CCLWNHS conducted in 2016–2019. The National Institute of Nutrition and Health (NINH), Chinese Center of Disease Control and Prevention (China CDC) organized the survey. To sample the participants, a multi-stage, stratified, cluster random sampling design was used to choose the participants in four provinces, Shandong, Guizhou, Jiangsu, and Hebei, which are considered eastern, western, southern, and northern parts of China. These provinces represent the different levels of economy and food cultures and give a valid sample of the dietary habits and nutritional health conditions of children and adolescents across the nation. All subjects underwent face-to-face interviews, physical check-ups, laboratory tests, and dietary check-ups. The process of data collection involved the use of standardized instruments and operating procedures to achieve data quality and reliability (25). The research was conducted in accordance with the ethical principles, and the informed consent was signed by the participants and their guardian. The Ethics Review Committee of China CDC approved the protocol (Approval No. 201614).

The first group consisted of 12,976 children and adolescents aged 6–18 years. Participants were eliminated in sequential order according to the pre-defined criteria: participants who did not fit within the 6–18 age range (*n* = 20), who did not have any height or weight measured (*n* = 3), who did not have complete dietary data (*n* = 124), and those whose energy intake values fell outside the 2.5th–97.5th percentile range of their respective age and sex groups (*n* = 642). These criteria resulted in the final analysis of 12,187 participants.

### 2.2 Assessment of dietary intake

Trained interviewers measured the dietary data through a validated food frequency questionnaire (FFQ). The FFQ is a systematically designed questionnaire developed by a group of nutritionists at China CDC, covering 12 food categories and 59 subcategories including staple foods, legumes, vegetables, fungi and algae, fruits, dairy products, meats, aquatic products, eggs, beverages, nutrient supplements, and other foods, encompassing all major dietary categories of the Chinese population. It is used to record the frequency and the specific amounts of intake of a large variety of foods. It has been extensively applied and tested in a number of large-scale child nutrition studies in China (26–29). The survey was conducted using standardized survey kits whereby the frequency, quantity, and portion size of all the food items were recorded by the staff, and the questionnaires were filled under the supervision of the guardians of the participants. In order to enhance the validity of the condiment intake measure, a three-day weighing procedure was further adopted consecutively to determine the real consumption of edible oil, salt, monosodium glutamate, and other seasonings in homes and school cafeterias. For nutrient and amino acid calculations, the daily average intake of each food was first calculated based on the frequency and portion size recorded in the FFQ, then multiplied by the corresponding energy, macronutrient, and 20 amino acid content data from the Chinese Food Composition Table (6th Edition) (30), and finally summed across all food items to obtain the total daily intake of various amino acids for each individual. The entire process was strictly standardized to ensure the consistency and completeness of dietary data assessment.

Since the dietary amino acid intake was significantly correlated with the total energy intake, the study used the energy density technique to control the amino acid intakes by adjusting them to eliminate the possibility of energy confounding (31–34). The amino acid intake was calculated as grams per 1,000 kilocalories of energy intake (g/1,000 kcal) and this enabled the determination of the relative composition of amino acids in the total diet. This method separates the effect of total energy hence explaining the independent relationships between individual amino acids and health outcomes.

### 2.3 Anthropometric measurements and ascertainment of hypertension

Anthropometric measurements were done in accordance with the Chinese industry standard methods of anthropometric measures in health surveillance (WS/T 424–2013) (35). Each instrument was nationally metrologically certified. The height was measured using a metal stadiometer to the nearest 0.1 cm, waist circumference (WC) measured using a measuring tape to the nearest 0.1 cm and weight measured using an electronic scale to the nearest 0.05 kg. The body mass index (BMI) was determined as weight (kg)/height (m^2^). Hypertension was determined based on the Chinese national health industry standard reference of screening of elevated blood pressure among children and adolescents aged 7–18 years (36). High blood pressure was determined as systolic and/or diastolic blood pressure that fell at or above the 95th percentile (P95) by sex, age, and height.

### 2.4 Covariate assessment

This study has corrected a variety of possible confounding variables in the analysis of the relationship between amino acid intake and hypertension. The covariates were: (1) sociodemographic factors: sex, age, and type of school (primary or secondary). (2) Physical activity: whether the participants achieved the recommended moderate-vigorous physical activity (MVPA; yes/no) and the duration of their MVPA daily. (3) Weight condition: overweight/obesity condition, which is identified using sex- and age-specific BMI criteria (37), and central obesity, which is identified using sex- and age-specific WC criteria (38). (4) Macronutrient intake: protein, fat and carbohydrate daily intake, total energy and ratio of energy sources of protein, fat and carbohydrates. Variance inflation factor (VIF) was used to check collinearity before constructing the models. The finding showed that there was a high probability of multicollinearity among certain variables related to macronutrients. In order to prevent bias in the estimation of the models and to be reliable, carbohydrate intake, carbohydrate energy percentage, and total energy intake were not included in the adjusted models. It is necessary to mention that the data on such variables like sex, age, physical activity (including participation and duration) were gathered through self-report.

### 2.5 Machine learning model construction

This study used four machine learning models, namely extreme gradient boosting (XGBoost), light gradient boosting machine (LightGBM), naïve Bayes (NB), and neural networks (NN) to set up the analytical framework. All four models are suitable for binary outcome prediction, can handle continuous and categorical features, and were evaluated using the area under the curve (AUC). The main goal was to systematically detect important amino acid variables that are linked to hypertension risk. XGBoost and LightGBM are both ensemble learning techniques that offer a trade-off between predictive power and interpretability, and are compatible with SHAP-based feature importance analysis. NB was used as a computationally efficient baseline model to provide a simple point of comparison. In contrast, the NN was employed to investigate the predictive capabilities upper limit, using multi-layer nonlinear transformations. By comparing the performance of these models, the best approach was chosen to identify the core amino acid features that have the highest contribution to hypertension risk. The underlying principles and characteristics of each model are explained as follows:

XGBoost is an ensemble learning algorithm that is based on gradient boosting but instead of building decision trees to fit residuals, it builds them iteratively. It uses a second-order Taylor expansions to optimize the objective function, thereby enhancing computational efficiency as well as improving predictive performance. With the built-in regularization and feature importance evaluation capabilities, XGBoost provides a good balance between accuracy, speed, and interpretability, and has been widely used in medical risk prediction research (39, 40).

LightGBM is a very efficient gradient boosting algorithm created by Microsoft. It helps to increase the efficiency of training using techniques such as gradient-based one-side sampling (GOSS) and exclusive feature bundling (EFB). By using histogram-based decision trees and a leaf-wise tree growth strategy, LightGBM has a fast training speed and low memory usage, which makes it especially suitable for large-scale, high-dimensional data. Given that this study contains over 12,000 samples and 20 amino acid intake variables, the computational efficiency of LightGBM makes it a contender model (41, 42).

NB is a probabilistic classifier that is based on Bayes’ theorem and the assumption of conditional independence between features and classifies observations by estimating posterior probabilities. It provides very fast training and has a high degree of adaptability to small sample and high-dimensional data, so it is a widely used baseline model for comparison with more complex algorithms. In this work, it was used as a baseline to determine if more sophisticated models can provide significant improvements in predictive capabilities (43, 44).

NN automatically learns the complex data patterns through multi-layer nonlinear transformations and have the universal approximation capability, which makes them capable of modeling highly complex functional relationships. However, their black box nature reduces the level of interpretability, and they often need large datasets to reduce overfitting. In this study, the neural network model was used to examine the upper limit of predictive performance and compared with tree-based models to see if models like LightGBM had sufficiently captured the key underlying information (45, 46).

### 2.6 Statistical analyses

The chi-square test was used to compare categorical variables between groups. Continuous variables were checked on the normality and it was not found to be normally distributed, hence they are presented as median (interquartile range) [M (Q_1_, Q_3_)], and between-group test was done using Mann–Whitney *U* test (Wilcoxon rank-sum test).

Before making predictive models, the Boruta algorithm (100 iterations) using random forest was used to select features that are related to the risk of hypertension. The variables were sociodemographic variables, physical activity, anthropometric variables, macronutrient intake and the energy-adjusted intake (g/1,000 kcal) of 20 dietary amino acids. Boruta analysis showed that all amino acid variables were statistically significant predictors of hypertension and were kept to be used in further machine learning and logistic regression analysis.

The associations between individual amino acids and hypertension risk were assessed by multivariate logistic regression and odds ratios (ORs) and 95% confidence intervals (95% CIs) were calculated. The models were modified stepwise: Model 1: unadjusted. Model 2: adjusted to sex, age, school type, MVPA participation and duration, absolute protein and fat intake and the percentage of energy as protein and fat. Model 3: additional adjustment of overweight/obesity and central obesity status on the basis of Model 2.

Four machine learning models were developed to determine the predictive capacity of amino acids and other related predictors of hypertension: XGBoost, LightGBM, NB, and NN. The receiver operating characteristic (ROC) curves and the AUC were used to assess model performance. In the case of the most effective model, SHAP, which is a game-theoretic-based model, was used to measure the contribution of each feature to the prediction outcome and to the identification of important predictors.

The machine learning models contained such features: Sociodemographic characteristics: sex, age, school type. Physical activity: MVPA participation and duration. Anthropometrics: obesity/overweight and central obesity. Food: the total energy consumed, protein, fat, and carbohydrate intake, and the percentage of their energy. Amino acids include: isoleucine (Ile), leucine (Leu), lysine (Lys), serine (Ser), cysteine (Cys), tyrosine (Tyr), phenylalanine (Phe), threonine (Thr), glycine (Gly), valine (Val), arginine (Arg), histidine (His), alanine (Ala), aspartic acid (Asp), glutamic acid (Glu), methionine (Met), proline (Pro), tryptophan (Trp), as well as sulfur-containing amino acids (SAA) and aromatic amino acids (AAA).

The identification of key amino acids was done by combining the results of machine learning models and logistic regression. The sensitivity analyses were done in the following way: stratified analyses to investigate consistency of associations within subgroups. Interaction tests to determine possible effect modifiers. Restricted cubic spline (RCS) to assess dose–response relationships. The Akaike information criterion (AIC) was used to determine the optimal number of knots and nonlinearity was tested formally. All the models were adjusted to covariates that were added to the final logistic regression model.

All the statistical calculations were done in R software (4.3.1). R packages were used such as Boruta (feature selection), MatchIT (data matching), stats (basic statistics), forestplot (forest plots), car (collinearity diagnostics), xgboost, lightgbm, e1071 (XGBoost, LightGBM, naïve Bayes), nnet (neural networks), pROC (ROC analysis), rms (RCS analysis), etc. A two sided *p*-value less than 0.05 was considered to be statistically significant.

## 3 Results

### 3.1 Baseline characteristics of the study population

**Figure 1:**
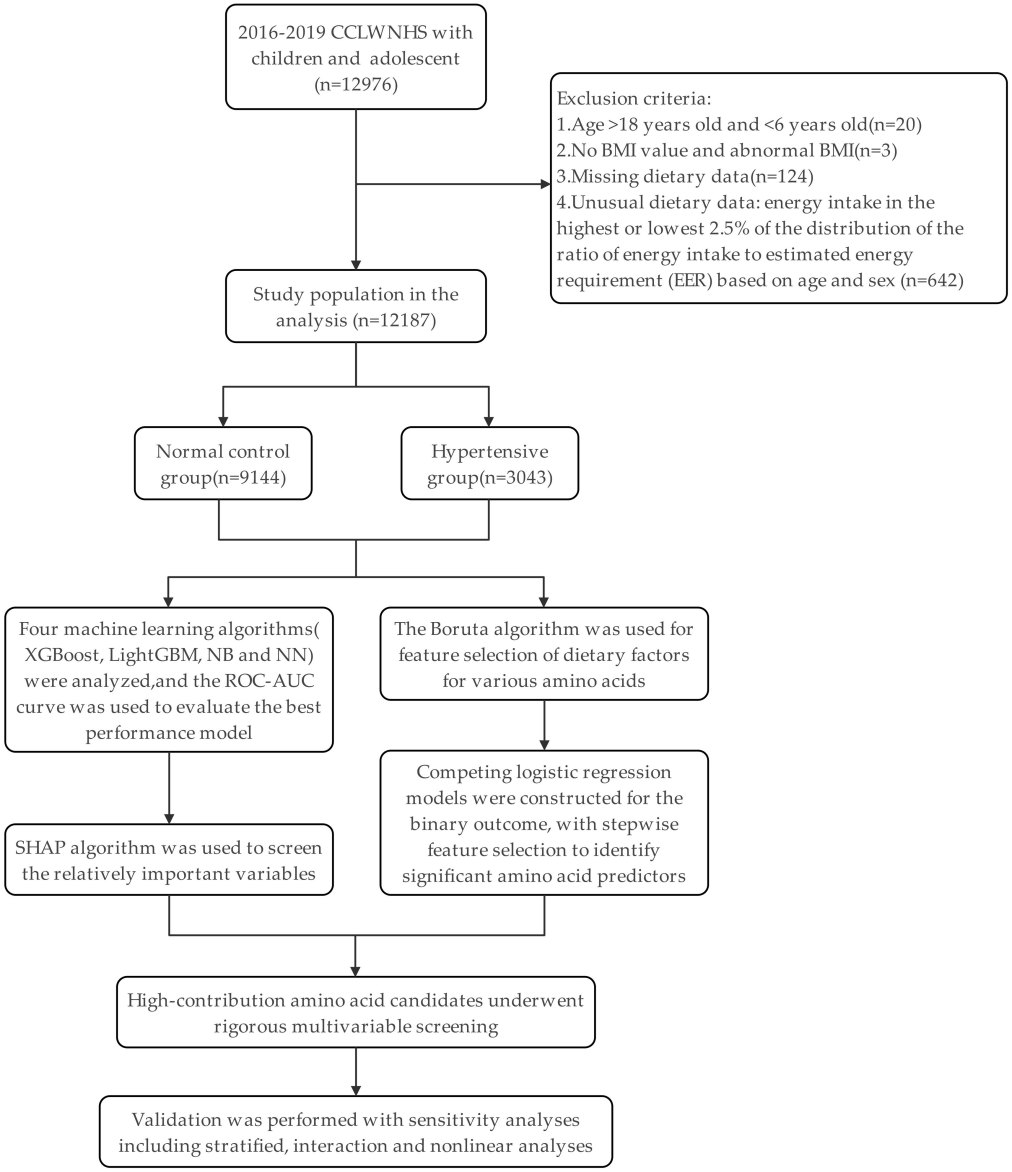
Flowchart of participant selection and statistical analysis.

**Figure 2:**
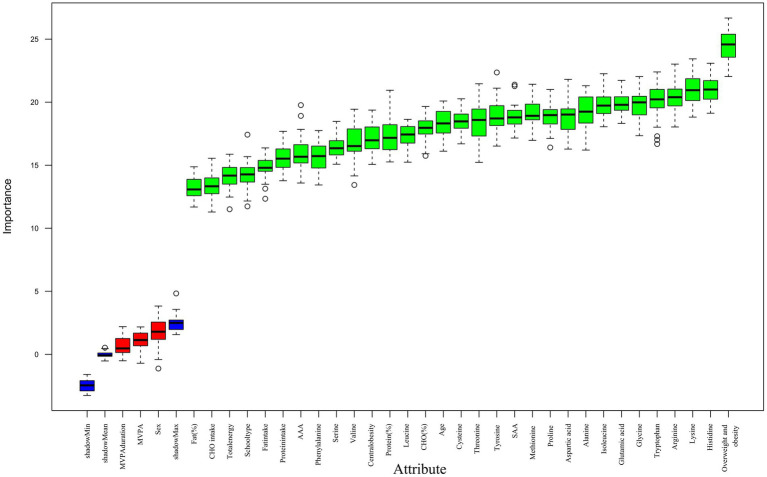
Boruta feature importance plot for variables associated with hypertension risk.

All participants were subsequently screened with 12,976 participants and 12,187 children and adolescents being included in the final analysis after the inclusion and exclusion criteria were applied (Figure 1). According to the Chinese screening criteria of hypertension in children and adolescents, with 95th percentile (P95) of blood pressure as the diagnostic cutoff, there were participants in a hypertension category (*n* = 3,043) and a control group of normotensive (*n* = 9,144). Figure 1 shows the screening flowchart. The Boruta feature selection method was used to validate the results and the results indicated that all the amino acid intake variables that were included in the analysis were statistically significant predictors of hypertension (Figure 2). The hypertension group was found to have a greater proportion of overweight/obesity and central obesity, and much higher body composition measurements such as systolic and diastolic blood pressure, BMI, WC and waist-height ratio as compared to the control group. As far as dietary intake is concerned, the hypertension group had much higher energy-adjusted intakes (g/1,000 kcal) of all amino acids than the control group. On the contrary, the intake of total energy or the three macronutrients showed no significant differences between groups (Table 1).

**Table 1:** Baseline features of the study population.

Parameters	Total (*n* = 12,187)	Normotensive control group (*n* = 9,144)	Hypertensive group (*n* = 3,043)	*p*-value
Male, *n* (%)	6,085 (49.93%)	4,588 (50.17%)	1,497 (49.19%)	0.360
Age (years)	11.00 (9.00, 14.00)	11.00 (9.00, 14.00)	11.00 (8.00, 13.00)	<0.001
MVPA (yes)	9,684 (79.46%)	7,291 (79.74%)	2,393 (78.64%)	0.204
MVPA duration (min)	30.00 (15.00, 60.00)	30.00 (15.00, 60.00)	30.00 (15.00, 60.00)	0.046
School type, *n* (%)				<0.001
Primary school	7,308 (59.97%)	5,317 (58.15%)	1,991 (65.43%)	
Secondary school	4,879 (40.03%)	3,827 (41.85%)	1,052 (34.57%)	
Systolic blood pressure (mmHg)	112.50 (104.67, 121.00)	109.00 (102.67, 115.67)	125.00 (118.67, 131.00)	<0.001
Diastolic blood pressure (mmHg)	66.67 (61.33, 72.67)	64.67 (60.00, 69.67)	75.00 (69.00, 80.67)	<0.001
Overweight and obesity, *n* (%)	3,213 (26.36%)	2042 (22.33%)	1,171 (38.48%)	<0.001
Central obesity, *n* (%)	2,188 (17.95%)	1,361 (14.88%)	827 (27.18%)	<0.001
BMI (kg/m^2^)	18.11 (16.00, 20.95)	17.93 (15.90, 20.56)	18.73 (16.31, 22.29)	<0.001
WC (cm)	62.90 (56.00, 70.20)	62.60 (55.75, 69.55)	63.50 (56.20, 73.25)	<0.001
WHtR	0.42 (0.40, 0.46)	0.42 (0.39, 0.45)	0.44 (0.41, 0.49)	<0.001
Protein (g/day)	103.55 (72.45, 146.24)	103.45 (72.45, 146.21)	103.69 (72.49, 146.48)	0.759
Fat (g/day)	33.32 (20.18, 54.56)	33.45 (20.23, 55.06)	32.96 (20.08, 53.42)	0.481
Carbohydrate (g/day)	304.18 (221.44, 420.47)	305.79 (222.88, 423.66)	300.20 (217.23, 413.23)	0.055
Total energy (kcal/day)	1976.68 (1443.13, 2756.81)	1987.29 (1449.74, 2769.74)	1941.85 (1429.36, 2708.58)	0.144
Protein percentage (%)	21.81 (18.49, 25.03)	21.75 (18.39, 24.98)	22.04 (18.83, 25.23)	0.001
Fat percentage (%)	15.39 (10.89, 21.20)	15.41 (10.86, 21.21)	15.36 (10.96, 21.11)	0.940
Carbohydrate percentage (%)	62.43 (56.31, 68.43)	62.49 (56.34, 68.52)	62.25 (56.21, 68.23)	0.129
Isoleucine (g/1,000 kcal)	1.95 (1.70, 2.21)	1.94 (1.69, 2.20)	1.97 (1.71, 2.24)	0.001
Leucine (g/1,000 kcal)	3.95 (3.35, 4.55)	3.94 (3.33, 4.54)	4.01 (3.41, 4.59)	0.001
Lysine (g/1,000 kcal)	2.57 (2.05, 3.10)	2.56 (2.05, 3.09)	2.60 (2.08, 3.14)	0.009
Serine (g/1,000 kcal)	2.36 (2.01, 2.70)	2.35 (1.99, 2.69)	2.39 (2.05, 2.72)	0.001
Cysteine (g/1,000 kcal)	0.66 (0.59, 0.75)	0.66 (0.58, 0.75)	0.67 (0.60, 0.76)	<0.001
Tyrosine (g/1,000 kcal)	1.74 (1.51, 1.99)	1.73 (1.51, 1.98)	1.76 (1.53, 2.00)	0.004
Phenylalanine (g/1,000 kcal)	2.40 (2.07, 2.71)	2.39 (2.06, 2.70)	2.43 (2.11, 2.74)	<0.001
Threonine (g/1,000 kcal)	2.03 (1.66, 2.40)	2.02 (1.65, 2.40)	2.06 (1.69, 2.42)	0.008
Glycine (g/1,000 kcal)	2.53 (2.08, 3.00)	2.53 (2.07, 2.99)	2.57 (2.10, 3.02)	0.004
Valine (g/1,000 kcal)	2.62 (2.18, 3.05)	2.60 (2.18, 3.05)	2.66 (2.22, 3.08)	0.003
Arginine (g/1,000 kcal)	3.07 (2.60, 3.56)	3.06 (2.59, 3.55)	3.10 (2.63, 3.59)	0.004
Histidine (g/1,000 kcal)	0.95 (0.81, 1.09)	0.94 (0.81, 1.09)	0.96 (0.82, 1.11)	<0.001
Alanine (g/1,000 kcal)	3.11 (2.49, 3.78)	3.10 (2.48, 3.78)	3.16 (2.54, 3.80)	0.016
Aspartic acid (g/1,000 kcal)	4.27 (3.54, 5.00)	4.26 (3.53, 4.99)	4.32 (3.58, 5.03)	0.007
Glutamic acid (g/1,000 kcal)	8.59 (7.37, 9.94)	8.53 (7.33, 9.88)	8.75 (7.52, 10.08)	<0.001
Methionine (g/1,000 kcal)	1.10 (0.88, 1.32)	1.10 (0.88, 1.32)	1.11 (0.90, 1.34)	0.006
Proline (g/1,000 kcal)	2.80 (2.33, 3.28)	2.78 (2.31, 3.27)	2.84 (2.40, 3.34)	<0.001
Tryptophan (g/1,000 kcal)	0.69 (0.58, 0.82)	0.69 (0.58, 0.82)	0.70 (0.58, 0.82)	0.036
Sulfur containing amino acids (g/1,000 kcal)	1.78 (1.51, 2.05)	1.78 (1.50, 2.04)	1.80 (1.54, 2.07)	<0.001
Aromatic amino acids (g/1,000 kcal)	4.13 (3.59, 4.69)	4.12 (3.57, 4.68)	4.18 (3.64, 4.74)	0.001

Continuous variables are shown as M (Q₁, Q₃); categorical variables are shown as the number of cases (percentage). WHtR, waist-to-height ratio.

### 3.2 Associations between amino acid intake and hypertension

Table 2 shows the findings of multivariate logistic regression analyses that were performed to establish the relationships between dietary amino acid consumption and the risk of hypertension among Chinese children and adolescents. Following the evaluation of multicollinearity, all the additional confounding variables were incorporated in the fully adjusted model (Model 3) that incorporated sex, age, MVPA participation and duration, school type, protein and fat intake and the proportion of their energy, overweight/obesity and central obesity status. In Model 3, a few amino acids were greatly related to hypertension. Cys (OR = 2.000, 95% CI: 1.350–2.967), Phe (OR = 1.649, 95% CI: 1.067–2.549), His (OR = 1.387, 95% CI: 1.026–1.874), and Pro (OR = 1.105, 95% CI: 1.026–1.188) were significantly positively associated with hypertension risk. Conversely, Thr (OR = 0.587, 95% CI: 0.413–0.836), Val (OR = 0.704, 95% CI: 0.526–0.941), Ala (OR = 0.809, 95% CI: 0.716–0.914) and Trp (OR = 0.516, 95% CI: 0.312–0.852) were protective factors against hypertension.

**Table 2:** Association between various dietary amino acids intake and the risk of hypertension in Chinese children and adolescents.

Amino acids	Model 1 OR (95% CI)	*p*	Model 2 OR (95% CI)	*p*	Model 3 OR (95% CI)	*p*
Isoleucine	1.170 (1.061–1.291)	0.002	1.106 (0.873–1.402)	0.404	1.205 (0.947–1.533)	0.129
Leucine	1.079 (1.032–1.127)	<0.001	1.044 (0.843–1.293)	0.694	1.055 (0.849–1.312)	0.628
Lysine	1.063 (1.011–1.118)	0.016	0.894 (0.776–1.030)	0.120	0.925 (0.801–1.068)	0.285
Serine	1.140 (1.055–1.231)	<0.001	1.186 (0.836–1.682)	0.338	1.226 (0.859–1.749)	0.261
Cysteine	1.901 (1.390–2.600)	<0.001	1.876 (1.272–2.767)	0.002	2.000 (1.350–2.967)	0.001
Tyrosine	1.149 (1.030–1.281)	0.013	0.845 (0.633–1.127)	0.251	0.947 (0.708–1.268)	0.716
Phenylalanine	1.164 (1.070–1.266)	<0.001	1.524 (0.993–2.341)	0.054	1.649 (1.067–2.549)	0.024
Threonine	1.099 (1.020–1.184)	0.013	0.588 (0.415–0.835)	0.003	0.587 (0.413–0.836)	0.003
Glycine	1.089 (1.029–1.152)	0.003	0.912 (0.781–1.064)	0.242	0.926 (0.790–1.085)	0.341
Valine	1.091 (1.024–1.162)	0.007	0.676 (0.507–0.901)	0.007	0.704 (0.526–0.941)	0.018
Arginine	1.081 (1.023–1.142)	0.006	0.924 (0.795–1.073)	0.298	0.972 (0.835–1.132)	0.716
Histidine	1.304 (1.109–1.532)	0.001	1.305 (0.970–1.755)	0.078	1.387 (1.026–1.874)	0.033
Alanine	1.049 (1.005–1.095)	0.030	0.815 (0.722–0.919)	0.001	0.809 (0.716–0.914)	0.001
Aspartic acid	1.049 (1.010–1.089)	0.014	0.868 (0.763–0.988)	0.032	0.906 (0.795–1.034)	0.142
Glutamic acid	1.052 (1.031–1.074)	<0.001	1.055 (1.027–1.082)	<0.001	1.052 (1.024–1.080)	<0.001
Methionine	1.184 (1.045–1.341)	0.008	0.769 (0.548–1.081)	0.131	0.782 (0.552–1.106)	0.164
Proline	1.124 (1.067–1.185)	<0.001	1.116 (1.039–1.199)	0.003	1.105 (1.027–1.188)	0.007
Tryptophan	1.211 (0.956–1.534)	0.113	0.486 (0.296–0.797)	0.004	0.516 (0.312–0.852)	0.010
Sulfur-containing amino acids	1.190 (1.077–1.315)	<0.001	1.148 (0.877–1.502)	0.315	1.201 (0.913–1.580)	0.190
Aromatic amino acids	1.080 (1.029–1.133)	0.002	1.009 (0.829–1.228)	0.932	1.082 (0.886–1.320)	0.440

Model 1 unadjusted. Model 2 was adjusted for sex (male or female), age (continuous), MVPA (yes or no), school type (primary school or secondary school), total MVPA duration (continuous), protein intake (continuous), fat intake (continuous), protein percentage (continuous), fat percentage (continuous). Model 3 was further adjusted for overweight and obesity (yes or no), central obesity (yes or no).

### 3.3 Machine learning-driven identification and sensitivity analysis of key amino acids

**Figure 3:**
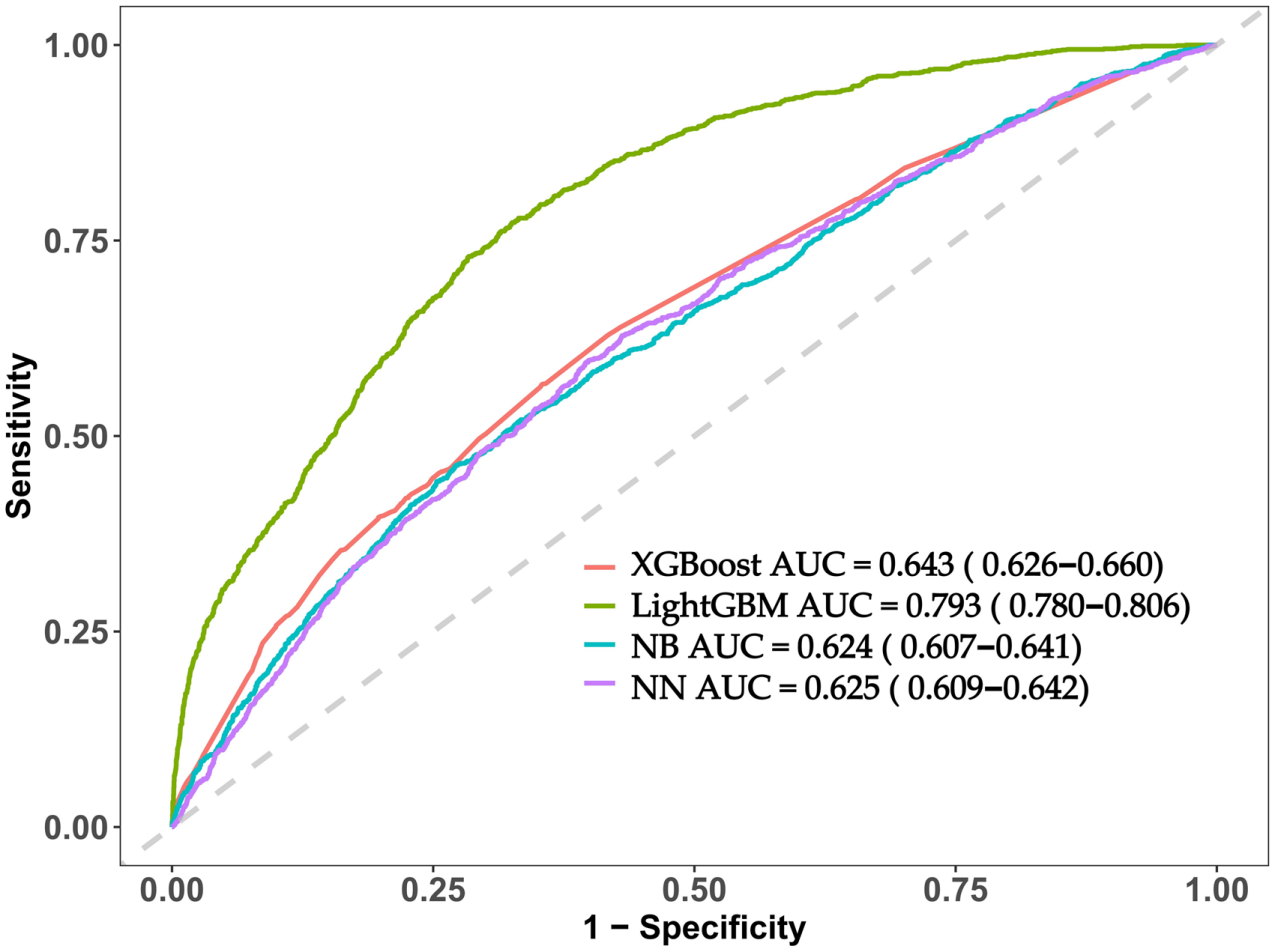
Predictive performance evaluation of ML-based predictive models using ROC curves.

**Figure 4:**
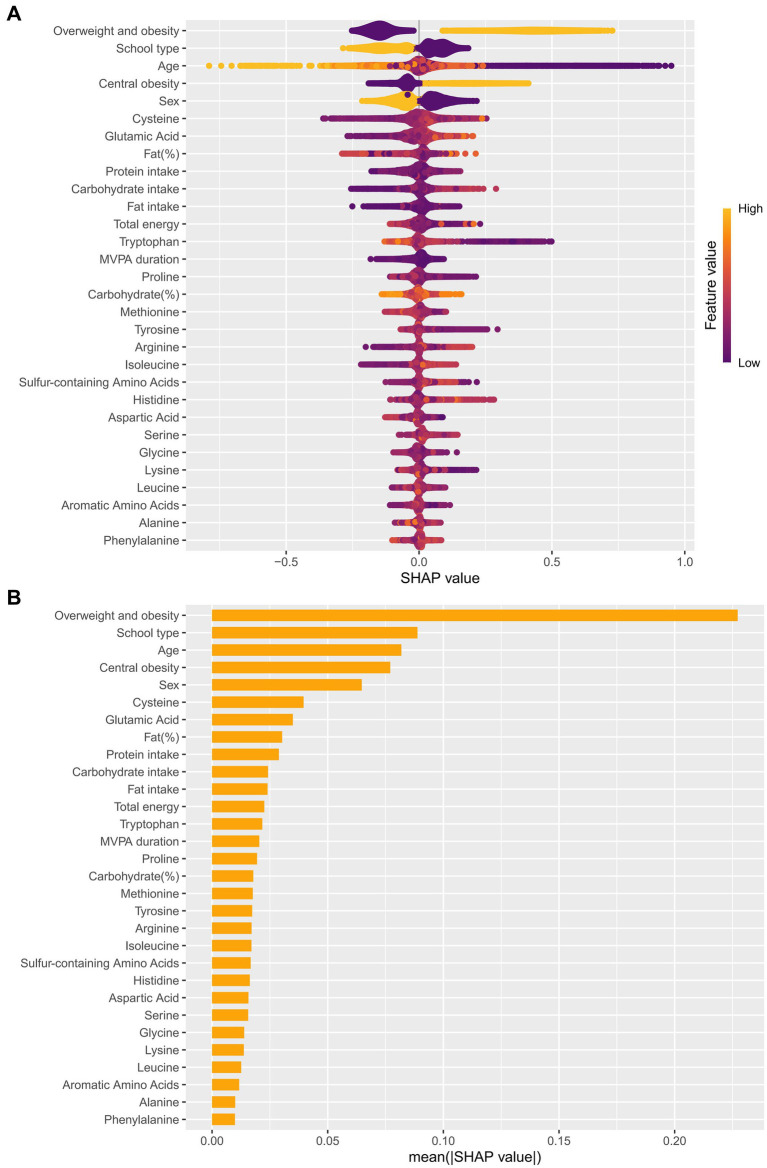
LightGBM model evaluation and interpretation via SHAP analysis. **(A)** Visualizing the direction and magnitude of each feature’s effect (purple: minimum feature range, yellow: maximum feature range). **(B)** Ranking of features by mean absolute SHAP value.

The ROC curves were used to evaluate the predictive performance of four machine learning algorithms in predicting hypertension incidence. The LightGBM model showed the highest performance, and the AUC was 0.793 (95% CI: 0.780–0.806) (Figure 3). The contribution of each feature to the risk of hypertension was systematically measured using the SHAP framework. Out of the top 15 core features that contributed to the risk of hypertension, a number of amino acids had significant contributions. The mean SHAP values were the highest in Cys and Glu which means that these amino acids were the main indicators of hypertension risk. The importance of Pro and Trp was also relatively high (Figure 4).

Cys, Glu, Trp, and Pro were found to be the important amino acids affecting the risk of hypertension by combining the feature importance ranking of the LightGBM model with the logistic regression outcomes (*p* < 0.05). Out of them, Cys, Glu and Pro had a positive relationship with hypertension, whereas Trp had a negative relationship.

**Figure 5:**
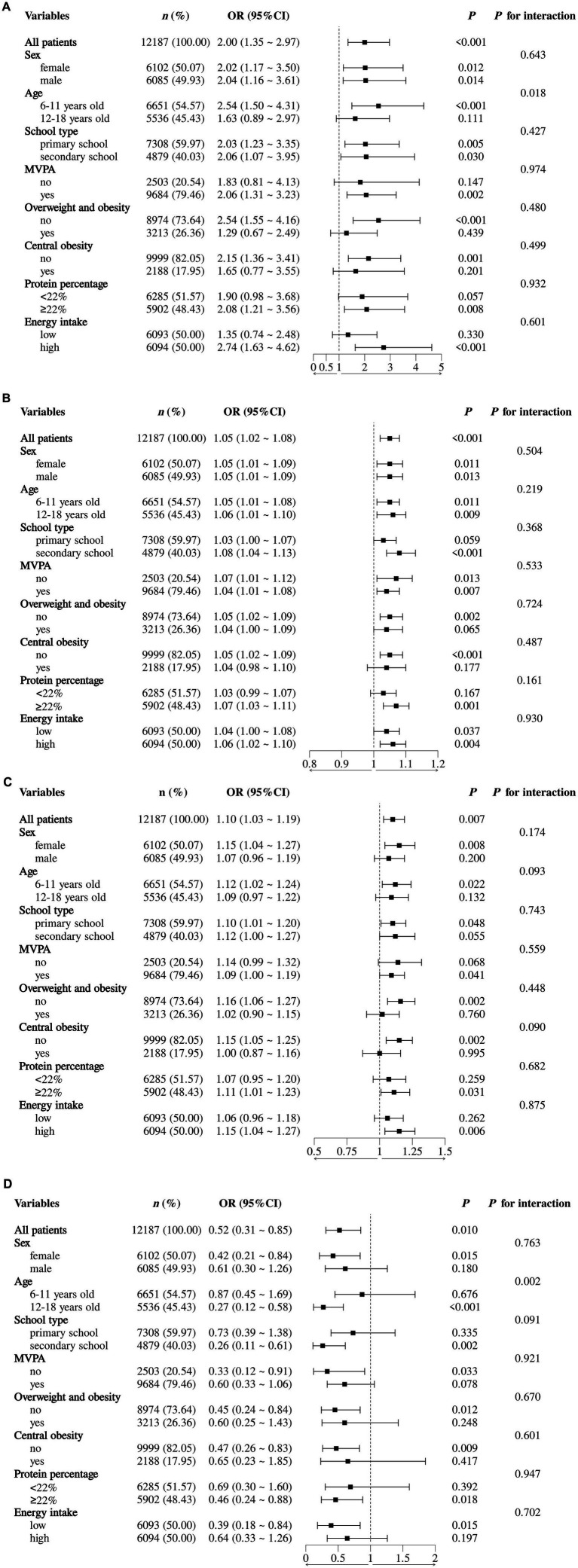
Stratified analysis forest plot of the association between dietary amino acids and hypertension risk. Amino acid species: **(A)** cysteine; **(B)** glutamic acid; **(C)** proline; **(D)** tryptophan. Statistical groupings were defined as follows: sex (male or female), age (6–11 years old or 12–18 years old), school type (primary school or secondary school), MVPA (yes or no), overweight and obesity (yes or no), central obesity (yes or no), protein percentage (<22% or ≥22%), energy intake (low or high). Regression models were adjusted for sex (male or female), age (continuous), MVPA (yes or no), school type (primary school or secondary school), total MVPA duration (continuous), protein intake (continuous), fat intake (continuous), protein percentage (continuous), fat percentage (continuous), overweight and obesity (yes or no), central obesity (yes or no).

Sensitivity analyses also investigated the correlation of these four amino acids with the risk of hypertension. Stratified analyses showed a stronger association of Glu, Pro and Trp in the secondary school group. Females had stronger associations with Pro and Trp. Conversely, the association of all four amino acids was found not to be significant at subgroups that had overweight/obesity, central obesity, and low protein intake. Risk-related amino acids (Cys and Pro) were found to have a greater positive correlation with hypertension among children aged 6–11 years and high-energy intake and those that met the recommendations of MVPA. On the other hand, the protective effect of the amino acid (Trp) was more evident in the participants with the age of 12–18 years, low energy intake, and participants who did not follow MVPA recommendations (Figure 5). Interaction tests revealed that possible confounding variables, such as age, sex, school-type, MVPA, overweight/obesity, central obesity, protein energy contribution ratio, and energy consumption, had no significant effects on the relationships between the four amino acids and hypertension risk (*p* > 0.05).

**Figure 6:**
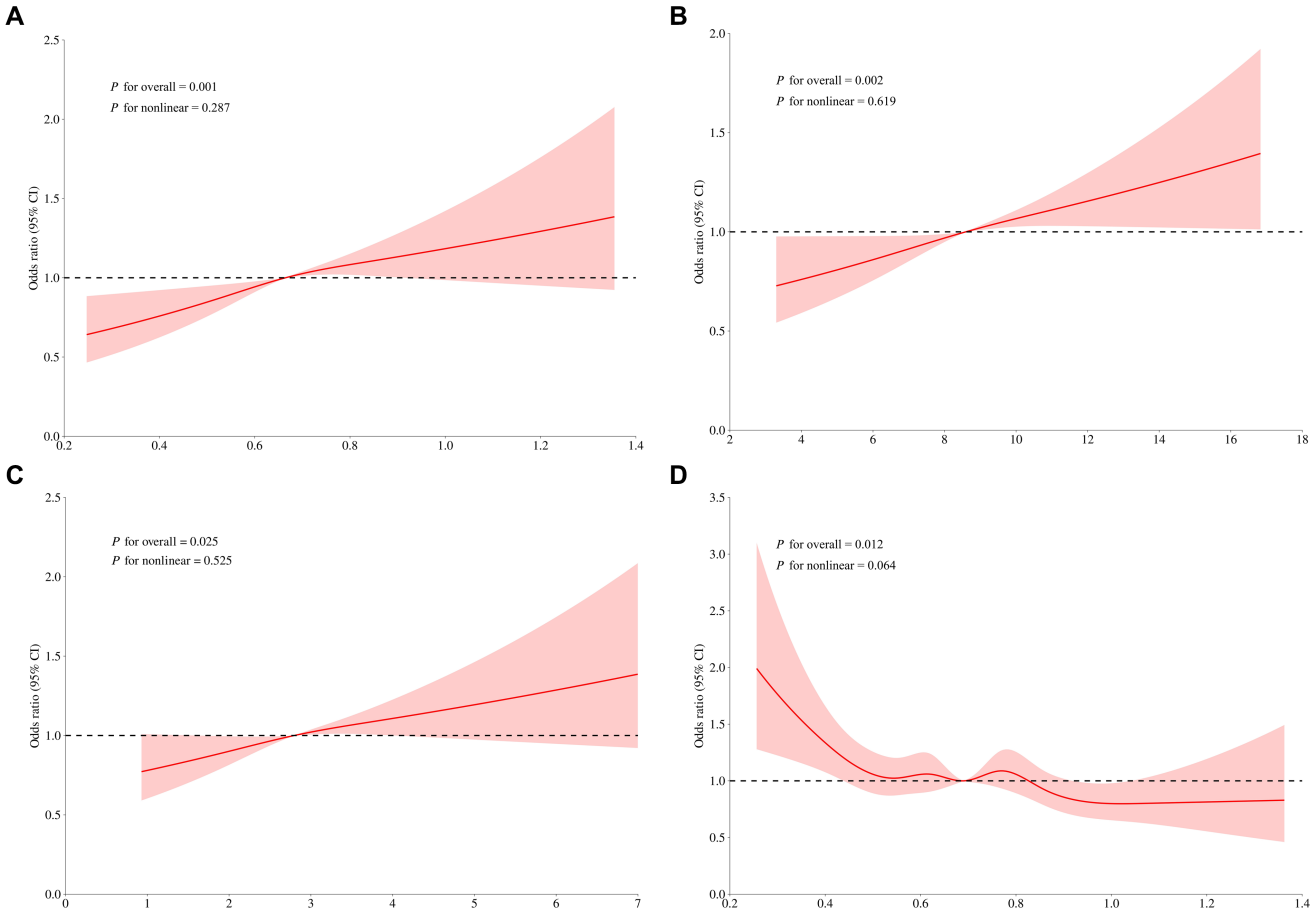
RCS analysis of different amino acid intake and the risk of hypertension. The model was adjusted for sex (male or female), age (continuous), MVPA (yes or no), school type (primary school or secondary school), total MVPA duration (continuous), protein intake (continuous), fat intake (continuous), protein percentage (continuous), fat percentage (continuous), overweight and obesity (yes or no), central obesity (yes or no). Amino acid species: **(A)** cysteine; **(B)** glutamic acid; **(C)** proline; **(D)** tryptophan.

This was found to be true in RCS analyses of the four amino acids (Cys, Glu, Trp, Pro) intake versus risk of hypertension, with no statistically significant nonlinearity (all *p* for nonlinearity > 0.05) (Figure 6).

## 4 Discussion

This study has systematically investigated the relationship between the risk of hypertension and the intake of dietary amino acids on a nationally representative sample of Chinese children and adolescents. The results show that there is a strong relationship between the relative consumption of various amino acids and hypertension. In particular, the positive relationships with hypertension risk were found with Cys, Glu, and Pro, and Trp was negatively correlated, which indicates that the mentioned amino acids could be significant factors influencing hypertension development and prevention in childhood and adolescence.

This work was the first to identify the major dietary amino acids that have a significant relationship with the risk of hypertension in a Chinese pediatric population in a systematic manner. Of these, Cys and Glu have been found to have the greatest predictive power in the LightGBM machine learning model and may therefore play a central role in the pathogenesis of hypertension. Cys is an amino acid with sulfur, and there are several lines of evidence for the adverse effect of Cys on blood pressure. Plasma Cys was found to have a positive relationship with blood pressure and was an independent risk factor of hypertension in Chinese adults (47, 48). High Cys levels were also found to be related to low postoperative blood pressure improvement following bariatric surgery in obese adolescents (49). Equally, high Cys levels in patients with obstructive sleep apnea were associated with high blood pressure, and these reduced with continuous positive airway pressure (CPAP) treatment (50). Experiments in animals have demonstrated that high-Cys diet of metabolic syndrome model rats, although not affecting body weight, can indirectly affect the maintenance of blood pressure by altering sulfur metabolism. There are a number of possible mechanisms that can explain the association between Cys and high blood pressure. To begin with, Cys may damage endothelial performance in the blood vessels. Being a pro-oxidative aminothiol, it causes redox imbalance, induces oxidative stress, stimulates inflammatory cascades like NF-κB, and blocks the production and use of nitric oxide. These effects affect the endothelium-dependent vasodilation, augment the vascular resistance, and enhance the rise of blood pressure (50, 51). Second, Cys may disrupt the sulfur metabolism, decreasing the synthesis of vascular protective factors, including taurine and hydrogen sulfide (18). Third, Cys could encourage obesity and lipid buildup, which worsens insulin resistance and indirectly leads to high blood pressure (52–57).

In the present research, a positive relationship was also found between the consumption of Glu and blood pressure. This correlation can be partially attributed to the fact that the system x_c_^−^ activity of the vascular endothelial cells is competitively inhibited by Glu, which inhibits the uptake of Cys, which subsequently accumulates more of Cys in plasma. This, in its turn, increases oxidative stress and endothelial dysfunction by the mechanisms mentioned above (50). Also, Glu can increase blood pressure by central and peripheral mechanisms. In the middle, overproduction of Glu may overstimulate N-methyl-D-aspartate (NMDA) receptors in the hypothalamic paraventricular nucleus and the rostral ventrolateral medulla (58, 59), disturbing the balance of inhibitory neurotransmitters, including gamma-aminobutyric acid (GABA), and increasing the transmission of sympathetic nerve impulses (60–62). At the periphery, Glu may cause oxidative stress and inflammation, inhibit vascular endothelial activity, decrease the production of nitric oxide (63), and directly activate the release of norepinephrine by sympathetic nerve endings. All these mechanisms lead to vasoconstriction, vascular resistance, and the ultimate high blood pressure (64).

Another finding of this research was that the intake of Pro was positively correlated with hypertension, which is in line with the results of the Tehran Lipid and Glucose Study (TLGS) cohort (65, 66), which also supports the conclusion that high Pro intake is likely to contribute to hypertension development. High plasma Pro levels have also been reported to be linked to high blood pressure (67). This association can be based on several pathophysiological mechanisms. First, Pro has the ability to regulate important effectors of the hippo signaling pathway, such as YAP (yes-associated protein) and TAZ (transcriptional coactivator with PDZ-binding motive), and stimulate Pro-glycine production in vascular fibroblasts. The process encourages excessive deposition of collagen in vascular wall, making the vascular wall stiffer (23, 68, 69). Second, Pro has the ability to mediate the activity of hypoxia-inducible factor-1α (HIF-1α), resulting in abnormal proliferation of vascular smooth muscle cells, thickening of the vascular wall, and remodeling, and hence increasing blood pressure (70). Also, insulin resistance is highly associated with high plasma Pro levels and can indirectly cause hypertension through metabolic dysregulation (71, 72).

This paper discovered that intake of Trp was strongly linked to a lower risk of hypertension, which is consistent with the results of the TwinsUK cohort nutritional study (73), which found an inverse relationship between Trp intake and systolic blood pressure and found that Trp is an independent nutrient in the regulation of blood pressure. There is growing evidence that Trp and its metabolites could be used as potential biomarkers and as therapeutic targets of hypertension (74). The protective effects of Trp could be explained by several mechanisms. The Trp-kynurenine metabolic pathway is activated to produce kynurenine (KYN) and xanthurenic acid capable of directly signaling the cGMP/cAMP pathway and Kv7 potassium channels in vascular smooth muscle, resulting in vasodilation. These metabolites also increase endothelial nitric oxide synthase (eNOS) to increase nitric oxide-mediated vasodilation. Kynurenic acid (KYNA) is an endogenous excitatory amino acid (EAA) receptor antagonist that inhibits sympathetic nerve excitation and blood pressure by activating the cardiovascular regulatory center in the rostral ventrolateral medulla (75, 76). Gut microbiota metabolites of Trp, such as indole-3-propionic acid (IPA) and indole-3-aldehyde, have the ability to activate the aryl hydrocarbon receptor (AHR) signaling pathway to attain better vascular endothelial functions and endothelium-dependent vasodilation. The same metabolites stimulate macrophages to express the anti-inflammatory cytokine IL-10 and prevent pro-inflammatory cytokines including TNF-α and minimizing vascular inflammation. They also increase antioxidant potential in the lung tissue, reducing the damage of the vascular endothelium caused by oxidative stress, and preserving the proper functioning of the vascular dilation (77, 78).

Interestingly, the RCS analysis in the present study failed to identify the nonlinear association between the four main amino acids (Cys, Glu, Pro, and Trp) and hypertension risk, which indicates that their influences were more or less linear within the given range of intake. Stratified analyses also showed that some of the amino acid associations, especially Trp and Pro, were stronger in females, which may indicate that sex can mediate the association between amino acids and blood pressure, and this may be because sex hormones may alter the metabolic pathways of the amino acids. The correlations of these amino acids with hypertension were not statistically significant among the obese population. This could be an indication of a masking effect of obesity, which is a strong state of metabolic disorder. The independent effect of dietary amino acids in obese children and adolescents might be less than the effect of obesity on blood pressure, thus undermining the independent effect of dietary amino acids. On the same grounds, the associations were not significant in participants that consumed low protein intake. This could be due to the fact that the blood pressure-regulating properties of amino acids are concentration-dependent, where in the case of a low-protein diet, the levels of intake might not be sufficient to induce the activity of these regulatory mechanisms, minimizing the protective and adverse effects. In addition, the low-protein diets are frequently combined with a high-carbohydrate energy supply pattern that may directly raise the blood pressure via such mechanisms as elevated blood glucose and insulin resistance. The independent relations between amino acids and hypertension may be lost or watered down by the strong hypertensive effect of such dietary patterns.

This study, in comparison to the previous ones where the primary attention was paid to adults or Western population, provides evidence on the correlation between dietary amino acids and hypertension in children and adolescents, which has specific dietary habits and metabolic peculiarities of the Chinese population. Our findings have several strengths, such as a large, nationally representative sample; a high level of control of the potential confounders; a dual-validation methodology that combines the traditional statistical analysis with machine learning; and a thorough sensitivity and dose–response analysis. There are, however, certain limitations that must be considered. First, the cross-sectional design does not allow making causal inferences, and the prospective cohort or intervention study is required to validate it further. Second, food intake was measured with food frequency questionnaires, which, despite being validated and complemented with a weighing method to enhance the accuracy, are also subject to recall bias and measurement error. Lastly, the paper has not reported blood amino acid concentrations or metabolomic profiling of the study, which would have provided direct biological validation of the proposed mechanistic pathways. We plan to use more objective methods of dietary assessment, in combination with measurements of biomarkers (e.g., plasma amino acid concentrations and indicators of metabolites), in order to minimize recall bias and clarify the molecular mechanisms by which amino acids affect blood pressure. Furthermore, multi-dimensional factors such as genetic background, gut microbiota, family and school environments, etc., will be combined to create a more comprehensive hypertension risk prediction model, which will enable the practical use of machine learning in cardiovascular health screening among children and adolescents. In addition, based on the important amino acid targets identified in this study, we hope to design and validate feasible dietary intervention strategies, and to test the effects of amino acid restriction or supplementation on blood pressure in children and adolescents.

## 5 Conclusion

This study presents initial data of independent relationships between various dietary amino acids and the risk of hypertension in Chinese children and adolescents. Cys, Glu, Pro, and Trp are identified as the focus of intervention. For children at high risk of hypertension, it is recommended to appropriately limit foods rich in Cys, Glu, and Pro (such as certain meats and condiments high in monosodium glutamate), while increasing the intake of Trp-rich foods (including dairy products, legumes, nuts, etc.) to optimize dietary protein structure. School meal programs may also benefit from these findings by adjusting ingredient ratios to meet children’s growth and development needs while reducing hypertension risk. Furthermore, this study provides evidence-based support for the refined revision of dietary guidelines for children, suggesting that the concept of amino acid balance should be incorporated to guide families in scientifically combining protein sources. Overall, these results provide a scientific foundation of precision nutrition programs to prevent hypertension in early life, while highlighting the public health significance of promoting balanced amino acid consumption and optimal overall dietary composition.

## Data Availability

The raw data supporting the conclusions of this article will be made available by the authors, without undue reservation.
